# Endovascular Treatment for Acute Isolated Internal Carotid Artery Occlusion: A Propensity Score Matched Multicenter Study

**DOI:** 10.1007/s00062-023-01342-7

**Published:** 2023-09-04

**Authors:** Daniel P. O. Kaiser, Tilman Reiff, Ulrich Mansmann, Daniela Schoene, Davide Strambo, Patrik Michel, Mohamad Abdalkader, Thanh N. Nguyen, Matthias Gawlitza, Markus A. Möhlenbruch, Peter A. Ringleb, Volker Puetz, Johannes C. Gerber, Simon Nagel

**Affiliations:** 1 Institute of Neuroradiology, Faculty of Medicine, University Hospital Carl Gustav Carus, Technische Universität Dresden, Fetscherstraße 74, 01307 Dresden, Germany; 2 Dresden Neurovascular Center, University Hospital Carl Gustav Carus, Dresden, Germany; 3 Department of Neurology, Heidelberg University Hospital, Heidelberg, Germany; 4 Institute of Medical Informatics, Biometry and Epidemiology, Ludwig-Maximilians-Universität München, Munich, Germany; 5 Department of Neurology, University Hospital Carl Gustav Carus, Dresden, Germany; 6 Department of Clinical Neurosciences, Lausanne University Hospital and University of Lausanne, Lausanne, Switzerland; 7 Department of Radiology, Boston Medical Center, Boston University Chobanian and Avedisian School of Medicine, Boston, MA USA; 8 Division of Interventional Neurology/Neuroradiology, Boston Medical Center, Boston University Chobanian and Avedisian School of Medicine, Boston, MA USA; 9 Department of Neuroradiology, University Hospital Leipzig, Leipzig, Germany; 10 Department of Neuroradiology, Heidelberg University Hospital, Heidelberg, Germany; 11 Department of Neurology, Klinikum Ludwigshafen, Ludwigshafen/Rhein, Germany

**Keywords:** Stroke, Intervention, Thrombectomy, Angiography, Cervical

## Abstract

**Purpose:**

The benefit of endovascular treatment (EVT) in patients with acute symptomatic isolated occlusion of the internal carotid artery (ICA) without involvement of the middle and anterior cerebral arteries is unclear. We aimed to compare clinical and safety outcomes of best medical treatment (BMT) versus EVT + BMT in patients with stroke due to isolated ICA occlusion.

**Methods:**

We conducted a retrospective multicenter study involving patients with isolated ICA occlusion between January 2016 and December 2020. We stratified patients by BMT versus EVT and matched the groups using propensity score matching (PSM). We assessed the effect of treatment strategy on favorable outcome (modified Rankin scale ≤ 2) 90 days after treatment and compared reduction in NIHSS score at discharge, rates of symptomatic intracranial hemorrhage (sICH) and 3‑month mortality.

**Results:**

In total, we included 149 patients with isolated ICA occlusion. To address imbalances, we matched 45 patients from each group using PSM. The rate of favorable outcomes at 90 days was 56% for EVT and 38% for BMT (odds ratio, OR 1.89, 95% confidence interval, CI 0.84–4.24; *p* = 0.12). Patients treated with EVT showed a median reduction in NIHSS score at discharge of 6 points compared to 1 point for BMT patients (*p* = 0.02). Rates of symptomatic intracranial hemorrhage (7% vs. 4%; *p* = 0.66) and 3‑month mortality (11% vs. 13%; *p* = 0.74) did not differ between treatment groups. Periprocedural complications of EVT with early neurological deterioration occurred in 7% of cases.

**Conclusion:**

Although the benefit on functional outcome did not reach statistical significance, the results for NIHSS score improvement, and safety support the use of EVT in patients with stroke due to isolated ICA occlusion.

**Supplementary Information:**

The online version of this article (10.1007/s00062-023-01342-7) contains supplementary material, which is available to authorized users.

## Introduction

Endovascular treatment (EVT) is the standard of care for patients with acute ischemic stroke (AIS) due to anterior circulation large vessel occlusion including intracranial internal carotid artery (ICA) and tandem occlusions [1, 2]. The term ICA occlusion encompasses two types of occlusions, ICA‑L and ICA‑T occlusions that affect the middle and anterior cerebral artery, respectively, as well as the rare isolated ICA occlusion, also known as ICA‑I occlusion. In the case of ICA‑I occlusion, antegrade collateral filling of the middle cerebral artery through external carotid artery collaterals or retrograde collateral filling via the circle of Willis can compensate for perfusion deficits. As these ICA occlusions have distinct pathophysiologies it is crucial to conduct a differentiated assessment of the effectiveness of EVT. Unfortunately, such an assessment was not performed in the individual patient data meta-analysis of the large randomized thrombectomy trials conducted by the HERMES collaboration [1]. Only four patients in MR CLEAN, one patient in REVASCAT and none in the ESCAPE trial presented with an isolated ICA occlusion [3–5]. No other randomized controlled trial focused on this subgroup.

The clinical presentation of stroke patients with a patent circle of Willis is highly variable [6]. In mildly or transiently affected patients, EVT and its associated risks must be weighed against the risk of best medical treatment (BMT) and the natural history of early neurologic deterioration or stroke recurrence [7].

Studies comparing EVT + BMT versus BMT in these patients are scarce or imbalanced [8–10]. Recently, one study investigated the benefit of initial BMT in patients with isolated ICA occlusion and reported a favorable outcome in 69% [11]. This rate is notably higher than that of 2 recent thrombectomy studies, which reported 34% and 26% favorable outcomes, respectively [12, 13]; however, this difference in outcomes is likely attributable to variations in the median baseline National Institutes of Health stroke scale (NIHSS) scores.

We aimed to compare outcomes of EVT + BMT (from here on only referred to as EVT) versus BMT alone in patients with acute isolated ICA occlusion in a multicenter study cohort with balanced groups. We hypothesized that EVT results in a greater likelihood of favorable clinical outcomes at 90 days compared with BMT and is safe in AIS patients with isolated ICA occlusion.

## Methods

### Study Design and Population

We conducted a retrospective multicenter observational study with propensity score matching and pooled data from the university hospitals of Dresden and Heidelberg, both Germany; Boston, MA, USA; and Lausanne, Switzerland. Between 1 January 2016 and 31 December 2020, we included consecutive patients with AIS due to symptomatic isolated occlusion of the ICA and no evidence of intracranial occlusion other than the ICA on noninvasive baseline imaging with computed tomography angiography (CTA) or magnetic resonance angiography (MRA). Eligible patients were adults with a NIHSS score ≥ 1 attributable to the vascular territory of the occluded ICA and treatment initiation within 24 h of stroke onset or last seen well. Additional inclusion parameters were prestroke functional independence with a modified Rankin scale (mRS) score of ≤ 2, no extensive infarction with a baseline Alberta stroke program early CT score (ASPECTS) ≥ 6, and no intracranial hemorrhage on baseline imaging.

This study was approved by the Ethics Committee of the TU Dresden, Germany (EK 272072017) with waiver of informed consent. The study complied with the Declaration of Helsinki and the Strengthening the Reporting of Observational Studies in Epidemiology (STROBE) statement.

### Baseline Characteristics

We collected the following patient characteristics: age, sex, comorbidities and vascular risk factors, prior antithrombotic and statin medications, prestroke mRS, and NIHSS on admission. We recorded stroke onset or the time the patient was last seen well and whether patients were admitted directly or transferred from another hospital. We collected data on baseline imaging with CT/CTA or MRI/MRA. An isolated ICA occlusion was diagnosed using standard CTA or MRA and confirmed by angiography in case the patient received EVT. Perfusion imaging was optional. We evaluated infarct size (ASPECTS), the side and location of occlusion, the Tan collateral score (0–3) with scores of 2 and 3 indicating good collaterals [14].

### Treatment Procedures

After qualifying imaging, patients were treated with EVT or BMT alone including intravenous thrombolysis (IVT) and antiplatelet therapy, according to international guidelines. The criteria for selecting patients for EVT included clinical radiological mismatch with neurological symptoms and small infarct size on noncontrast CT. The treating physicians chose the treatment strategy and devices at their discretion, including aspiration and/or stent retriever thrombectomy for EVT. Carotid stent with or without angioplasty was utilized for treatment of an underlying high-grade ICA stenosis. We did not use embolic protection devices. Prior to angioplasty and/or stenting procedures, patients received an acetylsalicylic acid (ASA) loading dose. If no intracranial hemorrhage was detected on follow-up imaging, clopidogrel was additionally administered. Tirofiban was used as an alternative to ASA.

We recorded the quality of reperfusion with the expanded thrombolysis in cerebral ischemia (eTICI) scale. The blood pressure was managed according to international and local guidelines during and after the procedure. The patients were monitored on a stroke unit or neurological intensive care unit. First follow-up imaging was performed within 24 h.

### Outcome and Safety

The primary endpoint was favorable functional outcome (mRS ≤ 2) 90 days after treatment. We also analyzed NIHSS score at discharge and delta NIHSS (admission–discharge NIHSS scores).

Safety outcome measures were symptomatic intracranial hemorrhage, death, and serious complications of EVT. In detail, we recorded any complication of EVT that could be or was associated with less favorable neurological outcome and any type of intracranial hemorrhage at follow-up imaging. Distal thrombus migration was defined as evidence of secondarily downstream migration of the thrombus with occlusion of the M1 segment of the middle cerebral artery during EVT [15]. Symptomatic intracranial hemorrhage was defined as any bleeding at 24 h on follow-up imaging associated with neurological deterioration of ≥ 4 points NIHSS [16].

### Statistical Analysis

Analysis of the raw data was performed for categorical variables with the χ^2^-test and with Fisher’s exact test as appropriate. Continuous variables were analyzed with the Mann-Whitney U-test. Continuous variables were presented as median and interquartile range (IQR), categorical variables as absolute and relative frequencies. Propensity score matching (PSM) method was 1:1 nearest neighbor matching without replacement. The caliper was 0.2, the propensity score was estimated with logistic regression, the target estimand was the average treatment effect on the treated (ATT). Covariates with pronounced imbalance at baseline, significant association to outcome, and clinical relevance were included as variables to be balanced in the PSM model. The following covariates were selected for propensity score matching: NIHSS on admission, ipsilateral extracranial occlusion, age, hypercholesterolemia, history of stroke, prestroke mRS, sex, and atrial fibrillation. The degree of equal distribution of individual covariates before and after PSM was measured by the absolute standardized mean difference (SMD). A SMD < 0.1 was taken as the limit for a balanced matching. To account for matching, univariate conditional logistic regression was chosen as primary logistic model to estimate the treatment effect in the matched sample. In order not to miss any confounders of the analysis, the distribution of all parameters in the raw group and in the patients excluded by matching was analyzed for unequal distribution. The detailed method of PSM and sensitivity analysis is described in the supplemental material. For all statistical analyses, a *p*-value of < 0.05 was considered significant. Analyses were performed using STATA 17.0 (Stata Corp, College Station, TX, USA), StatXact 12.0 (Cytel, Cambridge, MA, USA) and R Statistical Software (version 4.2.1; R Foundation for Statistical Computing, Vienna, Austria).

## Results

### Raw Sample

Of 4635 patients treated with anterior circulation large vessel occlusion during the study period, 164 (4%) had an isolated symptomatic ICA occlusion. We excluded 2 patients in whom treatment initiation was beyond 24 h after symptom onset and 13 patients with a prestroke mRS > 2. Of the 149 included patients, 74 (50%) received EVT and BMT and 75 (50%) BMT only. Table 1 shows the baseline characteristics of the raw sample.

**Table 1:** Univariate analysis of baseline parameters of the raw (unmatched) cohort by treatment groups

	EVT *n* = 74	BMT *n* = 75	*p*-value
*Age (years)*	72 (61–80)	63 (54–76)	0.0122^a^
*Sex male*	47 (64%)	46 (61%)	0.78^c^
*Hypertension*	57 (77%)	49 (65%)	0.12^c^
*Atrial fibrillation*	21 (28%)	19 (25%)	0.68^c^
*Coronary heart disease*	18 (24%)	10 (13%)	0.09^c^
*Hypercholesterolemia*	41 (55%)	50 (67%)	0.16^c^
*Current smoker*	21 (28%)	20 (27%)	0.82^c^
*Peripheral artery disease*	11 (15%)	10 (13%)	0.79^c^
*Diabetes mellitus*	21 (28%)	18 (24%)	0.54^c^
*Dialysis*	4 (5%)	4 (5%)	1.00^c^
*History of stroke*	19 (26%)	14 (19%)	0.30^c^
*Platelet inhibitors*	25 (34%)	23 (31%)	0.68^c^
*Anticoagulants*	8 (11%)	7 (9%)	0.76^c^
*Statins*	23 (31%)	25 (33%)	0.77^c^
*Stroke onset witnessed*	28 (38%)	23 (31%)	0.36^c^
*Side of occlusion: right*	31 (42%)	30 (40%)	0.81^c^
*Prestroke mRS*	0 (0–1)	0 (0–1)	0.38^a^
0	44 (59%)	50 (67%)	0.66^b^
1	17 (23%)	14 (19%)	–
2	13 (18%)	11 (15%)	–
*NIHSS at admission*	13 (7–19)	6 (3–10)	< 0.0001^a^
*Direct admission mode*^d^	45 (61%)	66 (88%)	0.0001^c^
*Modality of first imaging*			
CT	66 (89%)	68 (91%)	0.76^c^
MRI	11 (15%)	7 (9%)	0.30^c^
*Ipsilateral extracranial occlusion*	50 (68%)	69 (92%)	0.0002^c^
*Ipsilateral intracranial occlusion*	72 (97%)	71 (95%)	0.68^b^
*Contralateral occlusion*	4 (5%)	1 (1%)	0.21^b^
*Contralateral stenosis >* *70%*	5 (7%)	7 (9%)	0.56^c^
*ASPECTS*	10 (8–10)	10 (9–10)	0.84^a^
10	47 (64%)	50 (67%)	0.58^b^
9	7 (9%)	7 (9%)	–
8	12 (16%)	6 (8%)	–
7	5 (7%)	7 (9%)	–
6	3 (4%)	5 (7%)	–
*ASPECTS ≥* *7*	71 (96%)	70 (93%)	0.72^b^
*Collateral score*^e^* (n* *=* *107)*	3 (2–3)	3 (3–3)	0.17^a^
*Collaterals: circle of Willis*	–	–	0.65^b^
None	7 (9%)	8 (11%)	–
Anterior communicating artery	46 (62%)	39 (52%)	–
Anterior + posterior communicating artery	18 (24%)	23 (31%)	–
Posterior communicating artery	3 (4%)	5 (7%)	–
*Perfusion imaging*	40 (54%)	52 (69%)	0.0550^c^
*Perfusion mismatch (58 missing/not valid)*	34 (46%)	35 (47%)	0.07^c^

Data are *n* (%) or median (interquartile range)

*EVT* endovascular therapy, *BMT* best medical treatment, *mRS* modified Rankin scale, *NIHSS* National Institutes of Health Stroke Scale, *CT* computed tomography, *MRI* magnetic resonance imaging, *ASPECTS* Alberta stroke program early CT score, *CI* confidence interval

^a^Wilcoxon rank-sum (Mann-Whitney) test, ^b^Fisher’s exact test, ^c^χ^2^-test, ^d^vs. drip and ship, ^e^collateral score: 0: no collateral filling; 1: ≤ 50% but > 0 of the occluded MCA territory; 2: > 50% but < 100% of the occluded MCA; 3: 100% collateral supply of the occluded MCA territory

### Matching

**Fig. 1:**
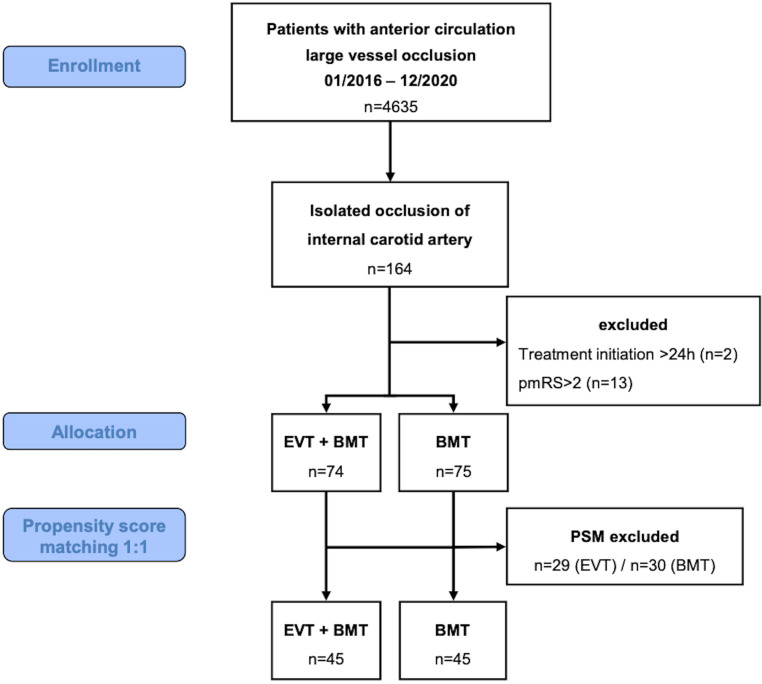
Flow chart of study design and patient selection. *EVT* endovascular treatment, *BMT* best medical treatment, *pmRS* prestroke modified Rankin Scale score, *PSM* propensity score matching

The number of observations was 149 in the original and 90 (45 in EVT and 45 in BMT) in the matched sample (Fig. 1). After PSM, a sufficient adjustment for the imbalanced covariates could be achieved (Table S2, Figure S1a–c). No covariate with a SMD > 0.1 after matching was significantly related to outcome. Detailed matching results and sensitivity analyses are described in the supplemental material.

### Baseline Characteristics

After matching, the median age was 65 years (IQR 56–78) and 54 (60%) patients were male. The median NIHSS score on admission was 9 (IQR 5–15) in the EVT and 9 (IQR 4–15) in the BMT group (*p* = 0.65). In the EVT group 31 (69%) patients were directly admitted to the tertiary hospital versus 39 (87%) patients in the BMT group (*p* = 0.07). The baseline characteristics of the matched sample are detailed in Table 2**.**

**Table 2:** Univariate analysis (matched sample) of the distribution of baseline parameters among treatment groups with conditional logistic regression. The odds ratio describes the allocation to the treatment group according to the factor assessed. An odds ratio of 1 indicates balanced groups

	EVT *n* = 45	BMT *n* = 45	Odds ratio	95% CI	*p*-value
*Age (years)*	66 (58–76)	65 (55–78)	1.00	0.97–1.04	0.91
*Male sex*	26 (58%)	28 (62%)	0.67	0.19–2.36	0.53
*Arterial hypertension*	34 (76%)	29 (64%)	1.71	0.67–4.35	0.26
*Atrial fibrillation*	12 (27%)	14 (31%)	0.82	0.34–1.97	0.66
*Coronary heart disease*	9 (20%)	8 (18%)	1.13	0.43–2.92	0.81
*Hypercholesterolemia*	24 (53%)	26 (58%)	0.82	0.34–1.97	0.66
*Current smoker*	13 (29%)	13 (29%)	1.00	0.40–2.52	1.00
*PAOD*	9 (20%)	5 (11%)	1.80	0.60–5.37	0.29
*Diabetes mellitus*	13 (29%)	9 (20%)	1.80	0.60–5.37	0.29
*Dialysis*	2 (4%)	3 (7%)	0.67	0.11–3.99	0.66
*History of stroke*	8 (18%)	7 (16%)	1.20	0.37–3.93	0.76
*Prior antithrombotic treatment*					
Platelet inhibitors	15 (33%)	14 (31%)	1.11	0.45–2.73	0.82
Anticoagulants	5 (11%)	5 (11%)	1.00	0.29–3.45	1.00
Statins	13 (29%)	13 (29%)	1.00	0.40–2.52	1.00
*Stroke onset witnessed*	16 (36%)	13 (29%)	1.43	0.54–3.75	0.47
*Side of occlusion: right*	20 (44%)	23 (51%)	0.77	0.33–1.75	0.53
*Prestroke mRS*	0 (0–1)	0 (0–1)	0.89	0.52–1.53	0.68
0	30 (67%)	28 (62%)	–	Base	–
1	8 (18%)	9 (20%)	0.84	0.28–2.52	0.76
2	7 (16%)	8 (18%)	0.82	0.25–2.70	0.75
*NIHSS on admission*	9 (5–15)	9 (4–15)	1.02	0.94–1.10	0.65
*Direct admission mode*^a^	31 (69%)	39 (87%)	0.38	0.14–1.08	0.07
*Ipsilateral extracranial occlusion*	39 (87%)	39 (87%)	1.00	0.29–3.45	1.00
*Ipsilateral intracranial occlusion*	43 (96%)	44 (98%)	0.50	0.05–5.51	0.57
*Contralateral occlusion*	2 (4%)	1 (2%)	2.00	0.18–22.06	0.57
*Contralateral stenosis >* *70%*	1 (2%)	6 (13%)	0.17	0.02–1.38	0.10
*ASPECTS ≥* *7*	43 (96%)	42 (93%)	1.50	0.25–8.98	0.66
*Collateral score (n* *=* *70)*	3 (2–3)	3 (2–3)	0.40	0.13–1.22	0.11
*Collaterals: circle of Willis*					
None	5 (11%)	4 (9%)	–	Base	–
Anterior communicating artery	28 (62%)	27 (60%)	0.82	0.22–3.08	0.76
Anterior + posterior communicating artery	10 (22%)	11 (24%)	0.75	0.17–3.34	0.71
Posterior communicating artery	2 (4%)	3 (7%)	0.54	0.06–5.05	0.59
*Perfusion imaging*	25 (56%)	32 (71%)	0.50	0.20–1.24	0.13
*Perfusion mismatch (34 missing/not valid)*	21 (47%)	21 (47%)	Na	Na	1.00

Data are median (IQR) or *n* (%)

*EVT* endovascular treatment, *BMT* best medical treatment, *CI* confidence interval, *mRS* modified Rankin scale, *NIHSS* National Institutes of Health stroke scale, *ASPECTS* Alberta stroke program early CT score, *PAOD* peripheral arterial occlusive disease, *Na* odds ratio and 95% CI in conditional logistic regression not valid

^a^vs. drip and ship

### Treatment Characteristics

Overall, 39 (43%) patients received IVT with a median time from onset to needle of 120 min (IQR 88–198). In the EVT group, 19 patients (42%) received IVT compared to 20 patients (44%) in BMT (*p* = 0.84). For EVT, the median time from onset to groin puncture was 360 min (IQR 249–615) and from door to groin puncture 94 min (IQR 65–173). Conscious sedation was the anesthesia modality in 62% of patients. Combination stent retriever and distal aspiration was utilized in 36% of patients; carotid stent with or without angioplasty was utilized for treatment of an underlying ICA stenosis in 49% of patients. Successful reperfusion (eTICI 2b–3) was achieved in 76% of the EVT patients. Details of procedural data are described in Table S4 of the supplemental material.

### Treatment Effect

**Fig. 2:**
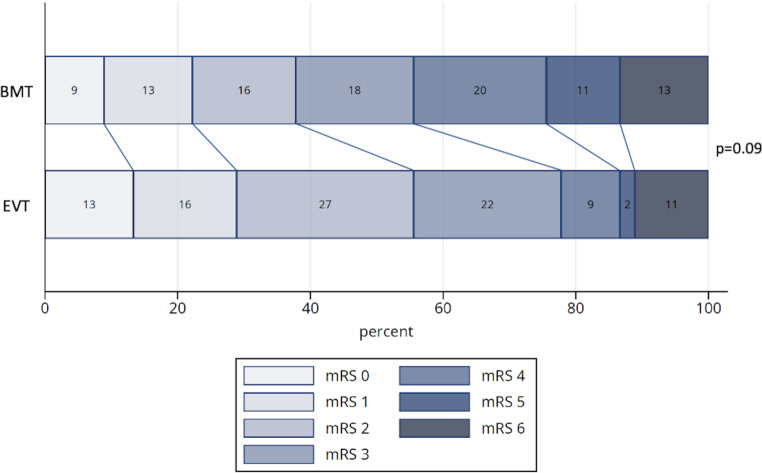
Modified Rankin scale (mRS) score 90 days after stroke by treatment group. Sample after propensity score matching. *BMT* best medical treatment (*n* = 45), *EVT* endovascular treatment (*n* = 45). Analysis with for matching stratified Wilcoxon-Mann-Whitney test

In the analysis of matched patients, the rate of favorable outcome (mRS ≤ 2 at 90 days) after treatment was 56% (*n* = 25) in the EVT and 38% (*n* = 17) in the BMT groups. In univariate conditional logistic regression, the likelihood of favorable outcome was not significantly higher for patients treated with EVT (OR 1.89; 95% CI 0.84–4.24; *p* = 0.12). Multivariate conditional logistic regression including all covariates that were unbalanced after matching (except collateral score due to partial lack of values) also showed no significant advantage of additional EVT treatment (OR 2.35; 95% CI 0.88–6.29; *p* = 0.09; Table 3) as well as the mRS levels of the EVT compared to the BMT group at day 90 after stroke (*p* = 0.09, Fig. 2). Median NIHSS at discharge was significantly lower in the EVT group (4, IQR 1–7) than in the BMT group (7, IQR 3–20; OR 0.95; 95% CI 0.90–1.00; *p* = 0.04). Patients treated with EVT also showed a better reduction in NIHSS score when comparing admission and discharge scores (median reduction of 6 vs. 1 NIHSS points; *p* = 0.02). The ASPECTS score after treatment was higher in the EVT group (median 8, IQR 7–10) compared to BMT (8, IQR 5–8; *p* = 0.05; Table S4).

**Table 3:** Analysis of treatment effect on favorable outcome (modified Rankin scale ≤ 2 after 90 days): univariate and multivariate conditional logistic regression of propensity score matched sample including imbalanced covariates with SMD > 0.1

	Odds ratio	95% CI	*p*-value
*Univariate analysis*			
Treatment group EVT vs. BMT	1.89	0.84–4.24	0.12
*Multivariate analysis*			
Treatment group EVT vs. BMT	2.35	0.88–6.29	0.09
Arterial hypertension	0.31	0.06–1.55	0.15
Contralateral ICA occlusion	0.70	0.04–11.32	0.80
Diabetes mellitus	0.58	0.12–2.82	0.50
PAOD	1.45	0.24–8.84	0.68

Due to partly missing values collateral score was not included in multivariate analysis

*SMD* absolute standardized mean difference, *CI* confidence interval, *EVT* endovascular treatment, *mRS* modified Rankin scale score, *ICA* internal carotid artery, *PAOD* peripheral arterial occlusive disease

### Safety

In the matched sample, the rate of symptomatic ICH was not significantly higher in the EVT compared with the BMT group (7%, *n* = 3 vs. 4%, *n* = 2; OR: 1.50; 95% CI 0.25–8.98; *p* = 0.66). The mortality rate at 90 days was comparable between both groups (EVT 11%, *n* = 5 versus BMT 13%, *n* = 6; OR 0.80; 95% CI 0.21–2.98; *p* = 0.74; Table S4).

In the raw sample, periprocedural complications occurred in 5 (11%) patients treated with EVT, of whom 3 (7%) had early neurological deterioration with an increased NIHSS of ≥ 4 points within 24 h. In detail, the complications were one distal thrombus migration, one arterial dissection, one extensive parenchymal hemorrhage, and one inadvertent stent retriever detachment. Multiple complications were reported in one patient.

## Discussion

In this study of patients with acute ischemic stroke due to symptomatic isolated ICA occlusion, we found that EVT was a safe and effective treatment option. We observed higher follow-up ASPECTS and significantly better results in the NIHSS score at discharge, which is a valid marker for the efficacy in patients treated with EVT; however, the 18% increase in good 90-day outcomes with EVT was not statistically significant.

Potential confounders with initial imbalance in the raw sample which were partially outcome relevant could be compensated by PSM. The exclusion of 59 patients in the matching process did not result in the selection of a collective that lacked essential characteristics of the predefined initial collective. Therefore, we do not consider the validity of the analysis with respect to the original overall collective to be limited. In addition, the sensitivity analysis with inverse probability of treatment weighting without exclusion of patients confirmed the result of the PSM.

Accounting for 4% of anterior large vessel occlusions, the rate of patients with isolated ICA occlusion treated in our centers was consistent with the MERCI and Multi-MERCI analyses but lower than the rate (7.8%) reported by the Italian Registry of Endovascular Treatment in Stroke [6, 13]. Compared with the MR CLEAN and REVASCAT trials [4, 5], these rates indicate the higher likelihood of facing a patient with AIS due to isolated ICA occlusion in a real-world setting.

Our study findings align with previously published research. A meta-analysis from 2021 found a higher probability of favorable outcome for EVT (OR 2.2, 95% CI 1.3–3.7) in an analysis of 7 studies [8]; however, the analysis was limited to isolated cervical ICA occlusion, and the low quality and number of studies precluded meta-regression analysis. Recently, an analysis of the MR CLEAN registry and MR CLEAN trial showed favorable outcomes in 54% of EVT-treated and 10% of non-EVT-treated patients with isolated distal ICA occlusion [9]; however, due to the low number of patients (*n* = 41 EVT and *n* = 10 non-EVT), no regression analyses or statistical tests were performed. Another recent study showed a good clinical outcome in 73% of patients treated with EVT compared with 61% of patients treated with BMT in a cohort of 73 patients (OR 1.7; 95% CI 0.64–4.6) [10]; however, only patients with isolated cervical ICA occlusion were included and the differences in baseline characteristics between treatment groups limited interpretation. Other studies focused only on one treatment strategy. Our study provides important information on the potential benefit of EVT in patients with isolated symptomatic ICA occlusion.

The median NIHSS score on admission in our cohort was 9. When treating these patients with moderate stroke symptoms, the main risk of EVT is neurological deterioration caused by complications, particularly due to distal thrombus migration. We found 5 (11%) patients with periprocedural EVT complications of whom only 1 (2%) had a distal thrombus migration. Other studies reported a thrombus migration rate of 18%, 20% and 22% during EVT in this setting [9, 15, 17]. Interestingly, in the Japanese study, [15] 8% (3/38) of patients also experienced a spontaneous thrombus migration without EVT and thrombus migration was associated with a significantly decreased likelihood of favorable outcome. Our data do not provide the opportunity to analyze the best EVT treatment strategy to prevent thrombus migration; however, it is worth noting that in our study, we predominantly used a combined approach utilizing stent retriever and aspiration thrombectomy, whereas the aforementioned studies with higher thrombus migration rates did not employ this combined approach.

A reported negative effect of intravenous thrombolysis on favorable outcome [18] was not shown in our study.

A recent study evaluated initial BMT in patients with minor stroke (median NIHSS score 3) and isolated ICA occlusion and reported a rate of 20% (11/56) neurological deterioration within the first week, 7% (4/56) rescue EVT but a high rate (68%; 38/56) of favorable outcomes at 90 days [11]. We found favorable outcomes in only 38% of the BMT patients. The lower rate may be explained by the higher median NIHSS score on admission, which is an independent predictor of less favorable outcome.

Differentiation of acute extracranial from intracranial carotid occlusion is limited on CTA and MRA, as absence of antegrade flow due to intracranial occlusion may result in lack of contrast opacification of proximal carotid segments and vice versa [19]. It could be hypothesized that digital subtraction angiography (DSA) results were taken into account and therefore pseudo-occlusions were less likely to remain undetected in the EVT than in BMT patients who did not receive DSA; however, the differences in involvement of extracranial ICA segments were levelled in the PSM.

Our study is limited by its retrospective design, lack of standardization, small sample size, and wide confidence interval in the primary endpoint, all of which may limit the interpretation of our findings.

## Conclusion

Although the benefit on functional outcome did not reach statistical significance, the results for NIHSS score improvement, higher follow-up ASPECTS, and safety support the use of EVT in patients with stroke due to isolated ICA occlusion. To enhance the reliability of assessing the safety and efficacy of EVT in this rare occlusion type, future studies should include a larger cohort of patients with isolated ICA occlusion.

### Supplementary Information

Additional information on propensity score matching, Figures S1a–c, Tables S1–S9. Alternate matching models, Figures S2–S3.

## References

[1] Goyal M, Menon BK, van Zwam WH (2016). Endovascular thrombectomy after large-vessel ischaemic stroke: a meta-analysis of individual patient data from five randomised trials. Lancet.

[2] Powers WJ, Rabinstein AA, Ackerson T (2019). Guidelines for the early management of patients with acute ischemic stroke: 2019 update to the 2018 guidelines for the early management of acute ischemic stroke: a guideline for healthcare professionals from the American Heart Association/American Stroke Association. Stroke.

[3] Goyal M, Demchuk AM, Menon BK (2015). Randomized assessment of rapid endovascular treatment of ischemic stroke. N Engl J Med.

[4] Berkhemer OA, Fransen PSS, Beumer D (2015). A randomized trial of intraarterial treatment for acute ischemic stroke. N Engl J Med.

[5] Jovin TG, Chamorro A, Cobo E (2015). Thrombectomy within 8 hours after symptom onset in ischemic stroke. N Engl J Med.

[6] Liebeskind DS, Flint AC, Budzik RF (2015). Carotid I’s, L’s and T’s: collaterals shape the outcome of intracranial carotid occlusion in acute ischemic stroke. J Neurointerv Surg.

[7] Khazaal O, Neale N, Acton EK (2022). Early neurologic deterioration with symptomatic isolated internal carotid artery occlusion: a cohort study, systematic review, and meta-analysis. Stroke Vasc Interv Neurol.

[8] Romoli M, Mosconi MG, Pierini P (2021). Reperfusion strategies in stroke due to isolated cervical internal carotid artery occlusion: systematic review and treatment comparison. Neurol Sci.

[9] Hoving JW, Kappelhof M, Schembri M (2021). Thrombectomy for acute ischemic stroke patients with isolated distal internal carotid artery occlusion: a retrospective observational study. Neuroradiology.

[10] Waters MJ, McMullan P, Mitchell PJ (2022). Endovascular therapy versus medical therapy for acute stroke attributable to isolated cervical internal carotid artery occlusion without intracranial large vessel occlusion. Stroke Vasc Interv Neurol.

[11] Ter Schiphorst A, Gaillard N, Dargazanli C (2021). Symptomatic isolated internal carotid artery occlusion with initial medical management: a monocentric cohort. J Neurol.

[12] Díaz-Pérez J, Parrilla G, Espinosa de Rueda M (2018). Mechanical thrombectomy in acute stroke due to carotid occlusion: a series of 153 consecutive patients. Cerebrovasc Dis.

[13] Sallustio F, Saia V, Marrama F (2021). Mechanical thrombectomy for acute intracranial carotid occlusion with patent intracranial arteries : the Italian registry of endovascular treatment in acute stroke. Clin Neuroradiol.

[14] Tan JC, Dillon WP, Liu S (2007). Systematic comparison of perfusion-CT and CT-angiography in acute stroke patients. Ann Neurol.

[15] Koge J, Matsumoto S, Nakahara I (2020). Impact of thrombus migration on clinical outcomes in patients with internal carotid artery occlusions and patent middle cerebral artery. J Neurol Sci.

[16] von Kummer R, Broderick JP, Campbell BCV (2015). The Heidelberg bleeding classification: classification of bleeding events after ischemic stroke and reperfusion therapy. Stroke.

[17] Jadhav A, Panczykowski D, Jumaa M (2018). Angioplasty and stenting for symptomatic extracranial non-tandem internal carotid artery occlusion. J NeuroIntervent Surg.

[18] Boulenoir N, Turc G, Henon H (2021). Early neurological deterioration following thrombolysis for minor stroke with isolated internal carotid artery occlusion. Eur J Neurol.

[19] Diouf A, Fahed R, Gaha M (2018). Cervical internal carotid occlusion versus pseudo-occlusion at CT angiography in the context of acute stroke: an accuracy, interobserver, and intraobserver agreement study. Radiology.

