# Supplementary material for: Endovascular Treatment for Acute Isolated Internal Carotid Artery Occlusion: A Propensity Score Matched Multicenter Study
Source: Clin Neuroradiol. 2023 Sep 4;34(1):125–33. doi: 10.1007/s00062-023-01342-7 (PMC10881648; doi:10.1007/s00062-023-01342-7)
Supplement: Supplementary file 1 — Additional information on propensity score matching, Figures S1a–c, Tables S1–S9. Alternate matching models, Figures S2–S3. [file 62_2023_1342_MOESM1_ESM.docx]

**Endovascular therapy for acute isolated internal carotid artery occlusion: a propensity score matched multicenter study**

**Supplemental Material**

**Method of propensity score matching**

Propensity score matching (PSM) was performed using the “MatchIt” command^1^ in R and is described as the model 1: Matching method was 1:1 nearest neighbor matching without replacement. The caliper was 0.2, the propensity score was estimated with logistic regression, the target estimand was the average treatment effect on the treated (ATT). Covariates with pronounced imbalance at baseline, significant association to outcome, and clinical relevance were included as variables to be balanced in the PSM model. Covariates only related to the EVT group were not included. We selected NIHSS on admission, ipsilateral extracranial occlusion, age, hypercholesterolemia, history of stroke, pre-stroke mRS, sex, and atrial fibrillation as covariates in PSM. For the sensitivity analysis, inverse probability treatment weighting (IPTW, method without exclusion of cases) was chosen as alternative matching model (model 2). The degree of equal distribution of individual covariates before and after PSM was measured by the absolute standardized mean difference (SMD). A SMD < 0.1 was taken as the limit for a balanced matching.^2^ To account for matching, univariate conditional logistic regression was chosen as primary logistic model to estimate the treatment effect in the matched sample.

As a sensitivity analysis, simple logistic regression adjusted for the propensity score was calculated alternatively. To control for imbalanced covariates after PSM, the treatment effect was tested with inclusion of covariates with a SMD ≥ 0.1 in a multivariate analysis using conditional logistic regression. Analysis for differences in mRS at day 90 after stroke between EVT and BMT groups was performed in the matched sample with for matching stratified Wilcoxon-Mann-Whitney test. Sensitivity analyses were performed using alternative methods of stratified analysis. In the logistic regression outcome models, only the treatment effect was interpreted as causal. An interpretation of the results of the covariates after PSM was not made because severely confounding cannot be excluded.^3^

**Results of propensity score matching**

Before matching, there was a pronounced imbalance especially of the clinically relevant covariates NIHSS on admission (13 [7–19] in EVT; 6 [3-10] in BMT; p<0.0001), age (72 [61-80] in EVT; 63 [54-76] in BMT; p=0.012) and ipsilateral extracranial carotid occlusion (EVT 50 [68%], BMT 69 [92%]; p=0.0002; **Table 1**). NIHSS on admission (OR 0.89; 95% CI 0.84–0.94; p<0.0001) and age (OR 0.96; 95% CI 0.94–0.99; p=0.0038) were significantly outcome related (**Table S1**). To correct for the imbalances of the raw sample, PSM was performed. The number of observations was 149 in the original and 90 (45 in EVT and 45 in BMT) in the matched sample. After PSM as described in the methods section (model 1), a sufficient adjustment for the most imbalanced covariates could be achieved (**Table S2, Figure S1a-c**). However, after matching, some covariates were still unbalanced: arterial hypertension, diabetes, peripheral arterial occlusive disease and the collateral score. No baseline covariate with a SMD >0.1 after matching was significant outcome related (**Table S5**). An analysis was performed to show differences between patients remaining in the sample after matching (n=90) and those excluded by matching (n=59): This showed that the proportion of patients with ipsilateral extracranial and ipsilateral intracranial occlusion was significantly higher in the matched sample (included sample 87%, excluded sample 69%; p=0.0106). Furthermore, in the matched sample less patients had a history of stroke (17% vs. 31%; p=0.0466) and more patients were treated with right-sided occlusions (48% vs. 31%; p=0.0360; **Table S3**).

**Sensitivity analyses**

When adding further covariates to matching model 1, only diabetes mellitus (referred to as model 3) showed a comparably good matching effect (**Table S6**). The sensitivity analysis with other matching models confirmed the analysis of the treatment effect with model 1 by similar results (e.g. for IPTW without exclusion of cases: OR 1.88; 95% CI 0.89-3.96; p=0.10; **Table S7** and **Figure S2** + **S3**). Sensitivity analysis controlling the multivariate conditional logistic regression was performed with propensity score stratified simple logistic regression. The results were inhomogeneous with no significance in univariate analysis but weak significance in multivariate analysis (OR 3.19, 95% CI 1.05-10.61; p=0.0471; **Table S8**). The sensitivity analysis to control the analysis of mRS levels confirmed the non-significant result (**Table S9**).

**Balance of propensity score before and after matching**

**Figure S1a:** Love plot of the absolute standardized mean differences (SMD) between endovascular therapy (EVT) and best medical treatment (BMT) before (raw) and after (matched) propensity score matching. ICA: internal carotid artery; mRS: modified Rankin Scale score; NIHSS: national institute of health stroke scale score; ASPECTS: Alberta stroke program early CT score; 1 collateral score: 0: no collateral filling; 1: ≤50% but >0 of the occluded MCA territory; 2: >50% but <100% of the occluded MCA; 3: 100% collateral supply of the occluded MCA territory; PAOD: peripheral arterial occlusive disease.

**Figure S1b:** Kernel density plot of propensity score before (raw) and after (matched) propensity score matching with model 1 illustrating balance between best medical treatment (BMT) and endovascular therapy (EVT). Raw sample n=149 (74 EVT, 75 BMT), matched sample n=90 (45 EVT, 45 BMT).

**Figure S1c:** Violin plot (boxplot & kernel density plot) + jitter plot of propensity score before (raw) and after (matched) propensity score matching with model 1. BMT: best medical treatment; EVT: endovascular therapy. Raw sample n=149 (74 EVT, 75 BMT), matched sample n=90 (45 EVT, 45 BMT).

**Raw sample: outcome relevance of covariates**

| **Table S1**: Association with favorable outcome (mRS ≤2 at day 90 after stroke) of imbalanced or relevant baseline covariates in raw sample.  The odds ratio describes the allocation to the outcome group according to the factor assessed. An odds ratio of 1 indicates balanced groups. | | | | | |
| --- | --- | --- | --- | --- | --- |
|  | **favorable outcome**  n=73 | **no favorable outcome**  n=76 | **odds ratio^1^** | **95% CI** | **p-value** |
| NIHSS on admission | 7 (4-9) | 13 (7-21) | 0.89 | 0.84 – 0.94 | <0.0001 |
| Extracranial occlusion | 56 (77%) | 63 (83%) | 0.68 | 0.30 – 1.52 | 0.35 |
| Age | 64 (54-74) | 72 (61-81) | 0.96 | 0.94 – 0.99 | 0.0038 |
| Collateral score^2^ (n=107) | 3 (3-3) | 3 (2-3) | 1.44 | 0.78 – 2.65 | 0.24 |
| Coronary heart disease | 10 (14%) | 18 (24%) | 0.51 | 0.22 – 1.20 | 0.12 |
| Arterial hypertension | 45 (62%) | 61 (80%) | 0.40 | 0.19 – 0.82 | 0.0134 |
| Hypercholesterolemia | 45 (62%) | 46 (61%) | 1.05 | 0.54 – 2.03 | 0.89 |
| Contralateral occlusion | 2 (3%) | 3 (4%) | 0.69 | 0.11 – 4.22 | 0.68 |
| History of stroke | 11 (15%) | 22 (29%) | 0.44 | 0.19 – 0.98 | 0.0444 |
| Pre-stroke mRS | 0 (0-1) | 0 (0-1) | 0.73 | 0.47 – 1.12 | 0.15 |
| Intravenous thrombolysis | 33 (45%) | 33 (43%) | 1.08 | 0.56 – 2.05 | 0.83 |
| ASPECTS ≥7 | 70 (96%) | 71 (93%) | 1.64 | 0.38 – 7.14 | 0.51 |
| Atrial fibrillation | 12 (16%) | 28 (37%) | 0.34 | 0.16 – 0.73 | 0.0060 |
| Sex (male) | 53 (73%) | 40 (53%) | 2.39 | 1.20 – 4.72 | 0.0127 |
| Side of occlusion: Right | 25 (34%) | 36 (47%) | 0.58 | 0.30 – 1.12 | 0.11 |
| Data are n (%) or median (interquartile range). mRS: modified Rankin scale; CI: confidence interval; NIHSS: National Institute of Health Stroke Scale; ^1^ logistic regression; ^2^ collateral score: 0: no collateral filling; 1: ≤50% but >0 of the occluded MCA territory; 2: >50% but <100% of the occluded MCA; 3: 100% collateral supply of the occluded MCA territory; ASPECTS: Alberta stroke program early CT score. | | | | | |

**Matched sample: balance of covariates**

| **Table S2:** Balance of covariates: Absolute standardized mean differences (SMD) between EVT and BMT before (raw) and after (matched) propensity score matching | | | | | | |
| --- | --- | --- | --- | --- | --- | --- |
|  | **raw** | | | **matched** | | |
|  | **EVT**  n=75 | **BMT**  n=74 | **SMD** | **EVT**  n=45 | **BMT**  n=45 | **SMD** |
| NIHSS on admission | 8.11 (6.43) | 13.20 (7.68) | **0.720** | 10.44 (6.98) | 10.96 (7.18) | 0.072 |
| Extracranial occlusion | 0.92 (0.27) | 0.68 (0.47) | **0.634** | 0.87 (0.34) | 0.87 (0.34) | <0.001 |
| Age | 64.91 (13.16) | 69.81 (12.62) | **0.380** | 66.27 (14.27) | 66.58 (12.73) | 0.023 |
| Collateral Score | 2.72 (0.55) | 2.50 (0.78) | **0.320** | 2.67 (0.57) | 2.41 (0.84) | **0.372** |
| Coronary heart disease | 0.13 (0.34) | 0.24 (0.43) | **0.282** | 0.18 (0.39) | 0.20 (0.40) | 0.056 |
| Arterial hypertension | 0.65 (0.48) | 0.77 (0.42) | **0.259** | 0.64 (0.48) | 0.76 (0.43) | **0.242** |
| Hypercholesterolemia | 0.67 (0.47) | 0.55 (0.50) | **0.231** | 0.58 (0.50) | 0.53 (0.50) | 0.089 |
| Contralateral occlusion | 0.01 (0.12) | 0.05 (0.23) | **0.226** | 0.02 (0.15) | 0.04 (0.21) | **0.123** |
| History of stroke | 0.19 (0.39) | 0.26 (0.44) | **0.168** | 0.16 (0.37) | 0.18 (0.39) | 0.059 |
| Pre-stroke mRS | 0.48 (0.74) | 0.58 (0.78) | **0.133** | 0.56 (0.78) | 0.49 (0.76) | 0.086 |
| Intravenous thrombolysis | 0.41 (0.50) | 0.47 (0.50) | **0.119** | 0.44 (0.50) | 0.42 (0.50) | 0.044 |
| ASPECTS ≥7 | 0.93 (0.25) | 0.96 (0.20) | **0.115** | 0.93 (0.25) | 0.96 (0.21) | 0.096 |
| Diabetes mellitus | 0.24 (0.43) | 0.28 (0.45) | 0.099 | 0.20 (0.40) | 0.29 (0.46) | **0.206** |
| Atrial fibrillation | 0.25 (0.44) | 0.28 (0.45) | 0.068 | 0.31 (0.47) | 0.27 (0.45) | 0.097 |
| Sex | 0.61 (0.49) | 0.64 (0.48) | 0.045 | 0.62 (0.49) | 0.58 (0.50) | 0.090 |
| PAOD | 0.13 (0.34) | 0.15 (0.36) | 0.044 | 0.11 (0.32) | 0.20 (0.40) | **0.244** |
| Current smoker | 0.27 (0.45) | 0.28 (0.45) | 0.038 | 0.29 (0.46) | 0.29 (0.46) | <0.001 |
| Dialysis | 0.05 (0.23) | 0.05 (0.23) | 0.003 | 0.07 (0.25) | 0.04 (0.21) | 0.096 |
| Data are standardized mean (standard deviation). EVT: endovascular therapy. BMT: best medical treatment. Bold font: imbalanced standardized mean difference (SMD) >0.1. ASPECTS: Alberta stroke program early CT score. mRS: modified Rankin Scale score. NIHSS: national institute of health stroke scale score. PAOD: peripheral arterial occlusive disease. | | | | | | |

**Differences of matched patients to patients excluded by propensity score matching**

| **Table S3**: Univariate analysis of baseline parameters of patients included and excluded in the propensity score matching sample. | | | |
| --- | --- | --- | --- |
|  | **included**  n=90 | **excluded**  n=59 | **p-value** |
| Age | 65 (56-78) | 71 (61-80) | 0.31^╧^ |
| Male sex | 54 (60%) | 39 (66%) | 0.45^ѱ^ |
| Arterial hypertension | 63 (70%) | 43 (73%) | 0.70^ѱ^ |
| Atrial fibrillation | 26 (29%) | 14 (24%) | 0.49^ѱ^ |
| Coronary heart disease | 17 (19%) | 11 (19%) | 0.97^ѱ^ |
| Hypercholesterolemia | 50 (56%) | 41 (69%) | 0.09^ѱ^ |
| Current smoker | 26 (29%) | 15 (25%) | 0.64^ѱ^ |
| Peripheral arterial occlusive disease | 14 (16%) | 7 (12%) | 0.53^ѱ^ |
| Diabetes mellitus | 22 (24%) | 17 (29%) | 0.55^ѱ^ |
| Dialysis | 5 (6%) | 3 (5%) | 1.00^ѱ^ |
| History of stroke | 15 (17%) | 18 (31%) | 0.0466^ѱ^ |
| Platelet inhibitors | 29 (32%) | 19 (32%) | 1.00^ѱ^ |
| Anticoagulants | 10 (11%) | 5 (8%) | 0.60^ѱ^ |
| Statins | 26 (29%) | 22 (37%) | 0.28^ѱ^ |
| Stroke onset witnessed | 29 (32%) | 22 (37%) | 0.52^ѱ^ |
| Side of occlusion: Right | 43 (48%) | 18 (31%) | 0.0360^ѱ^ |
| Pre-stroke mRS | 0 (0-1) | (0 (0-1) | 0.78^╧^ |
| NIHSS on admission | 9 (5-15) | 8 (4-16) | 0.48^╧^ |
| Direct admission mode^1^ | 70 (78%) | 41 (69%) | 0.26^ѱ^ |
| Ipsilateral extracranial occlusion | 78 (87%) | 41 (69%) | 0.0106^ѱ^ |
| Ipsilateral intracranial occlusion | 87 (97%) | 56 (95%) | 0.68^◊^ |
| Contralateral occlusion | 3 (3%) | 2 (3%) | 1.00^◊^ |
| Contralateral stenosis > 70% | 7 (8%) | 5 (8%) | 1.00^◊^ |
| ASPECTS ≥7 | 85 (94%) | 56 (95%) | 1.00^◊^ |
| Collateral score^2^ (n=107) | 3 (2-3) | 3 (3-3) | 0.13^╧^ |
| Collaterals: Circle of Willis |  |  | 0.54^◊^ |
| none | 9 (10%) | 6 (10%) |  |
| Anterior communicating artery | 55 (61%) | 30 (51%) |  |
| Anterior + posterior communicating artery | 21 (23%) | 20 (34%) |  |
| Posterior communicating artery | 5 (6%) | 3 (5%) |  |
| Perfusion imaging | 57 (63%) | 35 (59%) | 0.62^ѱ^ |
| Data are n (%) or median (interquartile range). ^╧^Wilcoxon rank-sum (Mann-Whitney) test; ^◊^Fisher's exact test; ^ѱ^Chi^2^ test. mRS: modified Rankin scale; NIHSS: National Institute of Health Stroke Scale; ASPECTS: Alberta stroke program early CT score; ^1^ vs. drip and ship; ^2^ 0: no collateral filling; 1: ≤50% but >0 of the occluded MCA territory; 2: >50% but <100% of the occluded MCA; 3: 100% collateral supply of the occluded MCA territory. | | | |

**Matched sample: treatment and outcome characteristics**

| **Table S4**: Univariate analysis (matched sample) of treatment characteristics, complications and outcome parameters stratified by treatment group with conditional logistic regression.  The odds ratio describes the allocation to the treatment group according to the factor assessed. An odds ratio of 1 indicates balanced groups. | | | | | |
| --- | --- | --- | --- | --- | --- |
|  | **EVT**  n=45 | **BMT**  n=45 | **odds ratio** | **95% CI** | **p-value** |
| Intravenous thrombolysis | 19 (42%) | 20 (44%) | 0.92 | 0.40 – 2.08 | 0.84 |
| Onset to needle time (min) | 115 (95-203) | 137 (86-193) | 1.00 | 0.98 – 1.02 | 0.81 |
| Onset to groin time (min) | 360 (249-615) | - | - | - | - |
| Door to needle time (min) | 29 (24-40) | 47 (37-69) | 0.91 | 0.81 – 1.03 | 0.15 |
| Door to groin time (min) | 94 (65-173) | - | - | - | - |
| Use of tirofiban | 3 (7%) |  |  |  |  |
| Device used for EVT |  |  |  |  |  |
| Stent retriever | 5 (11%) | - | - | - | - |
| Distal aspiration | 9 (20%) | - | - | - | - |
| Both | 16 (36%) | - | - | - | - |
| ICA angioplasty/stenting | 22 (49%) | - | - | - | - |
| Residual stenosis >50% if EVT | 12 (27%) | - | - | - | - |
| Type of anesthesia |  |  |  |  |  |
| Local | 2 (4%) | - | - | - | - |
| Sedation | 28 (62%) | - | - | - | - |
| General anesthesia | 14 (31%) | - | - | - | - |
| Periprocedural complications | 5 (11%) | - | - | - | - |
| eTICI score post intervention |  |  |  |  |  |
| 3 | 21 (47%) | - | - | - | - |
| 2c | 5 (11%) | - | - | - | - |
| 2b | 8 (18%) | - | - | - | - |
| 2a | 1 (2%) | - | - | - | - |
| 1 | 0 (0%) | - | - | - | - |
| 0 | 10 (22%) | - | - | - | - |
| Early neurological deterioration^1^ | 8 (18%) | 9 (20%) | 0.86 | 0.29 – 2.55 | 0.78 |
| Follow-up ASPECTS | 8 (7-10) | 8 (5-8) | 1.26 | 1.00 – 1.59 | 0.0534 |
| ICH on follow-up imaging | 5 (11%) | 4 (9%) | 1.25 | 0.34 – 4.66 | 0.74 |
| Symptomatic ICH | 3 (7%) | 2 (4%) | 1.50 | 0.25 – 8.98 | 0.66 |
| Type of ICH |  |  |  |  | ^ⱡ^ |
| SAH | 1 (2%) | 1 (2%) | - | - | - |
| HI1 | 0 (2%) | 3 (7%) | - | - | - |
| HI2 | 1 (2%) | 0 (0%) | - | - | - |
| PH1 | 0 (0%) | 0 (0%) | - | - | - |
| PH2 | 3 (7%) | 0 (0%) | - | - | - |
| In hospital mortality | 3 (7%) | 3 (7%) | 1.00 | 0.20 – 4.96 | 1.00 |
| Death at day 90 | 5 (11%) | 6 (13%) | 0.80 | 0.21 – 2.98 | 0.74 |
| NIHSS at discharge | 4 (1-7) | 7 (3-20) | 0.95 | 0.90 – 1.00 | 0.0383 |
| Delta NIHSS^2^ at discharge | 6 (0-9) | 1 (-2-4) | 1.08 | 1.01 – 1.15 | 0.0177 |
| Favorable outcome^3^ | 25 (56%) | 17 (38%) | 1.89 | 0.84 – 4.24 | 0.12 |
| mRS at D90 | 2 (1-3) | 3 (2-4) | 0.83 | 0.66 – 1.05 | 0.12 |
| mRS at D90 |  |  |  |  | 0.09**^†^** |
| 0 | 6 (13%) | 4 (9%) |  |  |  |
| 1 | 7 (16%) | 6 (13%) |  |  |  |
| 2 | 12 (27%) | 7 (16%) |  |  |  |
| 3 | 10 (22%) | 8 (18%) |  |  |  |
| 4 | 4 (9%) | 9 (20%) |  |  |  |
| 5 | 1 (2%) | 5 (11%) |  |  |  |
| 6 | 5 (11%) | 6 (13%) |  |  |  |
| Data are n (%) or median (interquartile range). EVT: endovascular therapy; BMT: best medical treatment; CI: confidence interval; mRS: modified Rankin scale; NIHSS: National Institute of Health Stroke Scale; ^1^ NIHSS increase ≥ 4 persisting for ≥ 24 hours; ^2^ NIHSS on admission minus NIHSS at discharge; ^3^ mRS ≤ 2 at day 90; ASPECTS: Alberta stroke program early CT score. CI: confidence interval; SAH: subarachnoid hemorrhage; HI: hemorrhagic infarction; PH: parenchymatous hematoma; ^ⱡ^ loglik converged prematurely; **^†^** stratified Wilcoxon-Mann-Whitney test. | | | | | |

**Matched sample: factors correlated with favorable outcome**

| **Table S5a**: Univariate analysis (matched sample) of baseline parameters stratified by favorable outcome (mRS D90 ≤ 2 after 90 days) with conditional logistic regression | | | | | |
| --- | --- | --- | --- | --- | --- |
|  | **favorable outcome**  n=42 | **no favorable outcome**  n=48 | **odds ratio** | **95% CI** | **p-value** |
| Age | 65 (54-74) | 68 (59-80) | 0.99 | 0.95 – 1.03 | 0.56 |
| Male sex | 30 (71%) | 24 (50%) | 0.87 | 0.37 – 10.92 | 0.42 |
| Arterial hypertension | 25 (60%) | 38 (79%) | 0.33 | 0.09 – 1.23 | 0.10 |
| Atrial fibrillation | 7 (17%) | 19 (40%) | 0.86 | 0.29 – 2.55 | 0.78 |
| Coronary heart disease | 6 (14) | 11 (23%) | 0.67 | 0.19 – 2.36 | 0.53 |
| Hypercholesterolemia | 22 (52%) | 28 (58%) | 1.20 | 0.37 – 3.93 | 0.76 |
| Current smoker | 13 (31%) | 13 (27%) | 0.83 | 0.25 – 2.73 | 0.76 |
| Peripheral arterial occlusive disease | 5 (12%) | 9 (19%) | 1.00 | 0.25 – 4.00 | 1.00 |
| Diabetes mellitus | 6 (14%) | 16 (33%) | 0.60 | 0.14 – 2.51 | 0.48 |
| Dialysis | 1 (2%) | 4 (8%) | 0.00 | 0.00 – inf | 1.00^2^ |
| History of stroke | 3 (7%) | 12 (25%) | 0.20 | 0.02 – 1.71 | 0.14 |
| Stroke onset witnessed | 15 (36%) | 14 (29%) | 1.67 | 0.40 – 6.97 | 0.48 |
| Side of occlusion: Right | 19 (45%) | 24 (50%) | 0.40 | 0.13 – 1.28 | 0.12 |
| Pre-stroke mRS | 0 (0-1) | 0 (0-1) | 0.67 | 0.34 – 1.34 | 0.26 |
| NIHSS on admission | 7 (4-9) | 13 (7-21) | 0.90 | 0.80 – 1.01 | 0.07 |
| Ipsilateral extracranial occlusion | 33 (79%) | 45 (94%) | - | - | - ^ⱡ^ |
| Ipsilateral intracranial occlusion | 42 (100%) | 45 (94%) | - | - | - ^ⱡ^ |
| Contralateral occlusion | 1 (2%) | 2 (4%) | 0.50 | 0.05 – 5.51 | 0.57 |
| ASPECTS ≥7 | 41 (98%) | 44 (92%) | 2.00 | 0.18– 22.06 | 0.57 |
| Collateral score^1^ (n=70) | 3 (2-3) | 3 (2-3) | 1.52 | 0.40 – 5.77 | 0.54 |
| Collaterals: Circle of Willis |  |  |  |  |  |
| none | 5 (12%) | 4 (8%) | 1.89 | 0.01 – 3.07 | 0.24 |
| Anterior communicating artery | 30 (71%) | 25 (52%) | 0.67 | 0.11 – 4.00 | 0.66 |
| Anterior + posterior communicating artery | 4 (10%) | 17 (35%) | 0.09 | 0.01 – 1.05 | 0.0551 |
| Posterior communicating artery | 3 (7%) | 2 (4%) |  | base |  |
| Data are n (%) or median (interquartile range); CI: confidence interval; mRS: modified Rankin Scale score; NIHSS: National Institute of Health Stroke Scale; ASPECTS: Alberta stroke program early CT score. ^1^ 0: no collateral filling; 1: ≤50% but >0 of the occluded MCA territory; 2: >50% but <100% of the occluded MCA; 3: 100% collateral supply of the occluded MCA territory; ^ⱡ^ loglik converged before variable, beta may be infinite. | | | | | |

| **Table S5b**: Univariate analysis (matched sample) of procedural parameters stratified by favorable outcome (mRS ≤ 2 after 90 days) with conditional logistic regression | | | | | |
| --- | --- | --- | --- | --- | --- |
|  | **favorable outcome**  n=42 | **no favorable outcome**  n=48 | **odds ratio** | **95% CI** | **p-value** |
| Intravenous thrombolysis | 18 (43%) | 21 (44%) | 0.56 | 0.19 – 1.66 | 0.29 |
| Treatment group EVT | 25 (60%) | 20 (42%) | 1.89 | 0.84 – 4.24 | 0.12 |
| Onset to needle time (min) | 115 (84-169) | 170 (90-210) | 1.02 | 0.97 – 1.07 | 0.49 |
| Onset to groin time (min) | 360 (211-627) | 345 (270-560) | - | - | -^2^ |
| Door to groin time (min) | 94 (68-173) | 111 (57-132) | - | - | -^2^ |
| Device used for EVT |  |  | - | - | -^2^ |
| Stent retriever | 3 (7%) | 2 (4%) |  |  |  |
| Distal aspiration | 6 (14%) | 3 (6%) |  |  |  |
| Both | 8 (19%) | 8 (17%) |  |  |  |
| ICA angioplasty/stenting | 13 (31%) | 9 (19%) | 1.00 | 0.14 – 7.10 | 1.00 |
| Residual stenosis >50% if EVT | 12 (29%) | 28 (58%) | 0.30 | 0.08 – 1.09 | 0.07 |
| Type of anesthesia |  |  | - | - | -^2^ |
| Local | 1 (2%) | 1 (2%) |  |  |  |
| Sedation | 18 (43%) | 10 (21%) |  |  |  |
| General anesthesia | 6 (14%) | 8 (17%) |  |  |  |
| Periprocedural complications | 2 (5%) | 3 (6%) | 0.67 | 0.11 – 4.00 | 0.66 |
| Early neurological deterioration^1^ | 4 (10%) | 13 (27%) | 0.14 | 0.02 – 1.16 | 0.07 |
| eTICI score post intervention |  |  | - | - | -^2^ |
| 3 | 15 (36%) | 8 (17%) |  |  |  |
| 2c | 2 (5%) | 3 (6%) |  |  |  |
| 2b | 5 (12%) | 3 (6%) |  |  |  |
| 2a | 0 (0%) | 1 (2%) |  |  |  |
| 1 | 0 (0%) | 0 (0%) |  |  |  |
| 0 | 4 (10%) | 6 (13%) |  |  |  |
| Follow-up ASPECTS | 8 (7-9) | 8 (4-8) | 1.53 | 0.99 – 2.36 | 0.0577 |
| ICH on follow-up | 3 (7%) | 6 (12%) | 0.40 | 0.08 – 2.06 | 0.27 |
| Symptomatic ICH | 0 (0%) | 5 (10%) | - | - | - ^ⱡ^ |
| Type of ICH |  |  |  |  | - ^ⱡ^ |
| SAH | 1 (2%) | 1 (2%) | - | - | - |
| HI1 | 0 (0%) | 3 (6%) | - | - | - |
| HI2 | 1 (2%) | 0 (0%) | - | - | - |
| PH1 | 0 (0%) | 0 (0%) | - | - | - |
| PH2 | 1 (2%) | 2 (4%) | - | - | - |
| Data are n (%) or median (interquartile range); CI: confidence interval; EVT: endovascular therapy; ICA: internal carotid artery; ICH: intracranial hemorrhage; SAH: subarachnoid hemorrhage; HI: hemorrhagic infarction; PH: parenchymatous hematoma; ASPECTS: Alberta stroke program early CT score; mTICI: modifed treatment in cerebral ischemia score; ^1^ increase in ≥4 points of the NIHSS within first 24h; ^2^ ran out of iterations and did not converge; ^ⱡ^ loglik converged before variable; beta may be infinite. | | | | | |

**Matching model 1 supplemented by further covariates**

| **Table S6:** Matching characteristics after adding further covariates with SMD^1^ >0.1 to propensity score matching model 1 | | | | |
| --- | --- | --- | --- | --- |
| **Added covariate** | **No. of matched cases** | **No. of SMD^1^ >0.1** | **mean SMD^1^** | **reduction of mean SMD^1^ (%)** |
| Model 1 | 45:45 | 5/18 | 0.111 | 49% |
| + arterial hypertension | 44:44 | 13/18 | 0.152 | 30% |
| + contralateral occlusion | 45:45 | 10/18 | 0.141 | 35% |
| + diabetes mellitus (= **model 3**) | 45:45 | 9/18 | 0.100 | 54% |
| + PAOD | 44:44 | 11/18 | 0.129 | 40% |
| + arterial hypertension | 43:43 | 13/18 | 0.168 | 22% |
| + contralateral occlusion |  |  |  |  |
| + diabetes mellitus |  |  |  |  |
| + PAOD |  |  |  |  |
| SMD: standardized mean difference. ^1^ matched sample. Covariate “collateral score” could not be included in propensity score matching model due to missing values in BMT cases; PAOD: Peripheral arterial occlusive disease. | | | | |

| **Table S7:** Sensitivity analysis of treatment effect on favorable outcome (mRS ≤2 after 90 days). Alternative method of analysis and overview of several matching models. | | | |
| --- | --- | --- | --- |
|  | **odds ratio** | **95% CI** | **p-value** |
| Univariate conditional logistic regression (**matching** **model 1**) |  |  |  |
| Treatment group EVT vs. BMT | 1.89 | 0.84 – 4.24 | 0.12 |
| Univariate logistic regression, stratified for propensity score (**matching model 1**) |  |  |  |
| Treatment group EVT vs. BMT | 2.17 | 0.93 – 5.22 | 0.08 |
| Propensity score | 0.16 | 0.02 – 1.44 | 0.11 |
| Univariate logistic regression (IPTW – **matching** **model 2**) |  |  |  |
| Treatment group EVT vs. BMT | 1.88 | 0.89 – 3.96 | 0.10 |
| Univariate conditional logistic regression (model 1 + diabetes mellitus = **matching model 3**) |  |  |  |
| Treatment group EVT vs. BMT | 2.14 | 0.87 – 5.26 | 0.10 |
| CI: confidence interval; EVT: endovascular therapy; BMT: best medical therapy; IPTW: Inverse probability of treatment weighting. | | | |

| **Table S8:** Sensitivity analysis of treatment effect on favorable outcome (mRS ≤2 after 90 days) with uni- and multivariate logistic regression stratified for propensity score (matched sample) including all imbalanced covariates with SMD >0.1 | | | |
| --- | --- | --- | --- |
|  | **odds ratio** | **95% CI** | **p-value** |
| Univariate analysis |  |  |  |
| Treatment group EVT vs. BMT | 2.17 | 0.93 – 5.22 | 0.08 |
| Propensity score | 0.16 | 0.02 – 1.44 | 0.11 |
| Multivariate analysis |  |  |  |
| Treatment group EVT vs. BMT | 3.19 | 1.05 – 10.61 | 0.0471 |
| Propensity score | 0.20 | 0.01 – 3.42 | 0.28 |
| Collateral score^1^ (n=70) | 1.30 | 0.54 – 3.30 | 0.57 |
| Arterial hypertension | 0.38 | 0.11 – 1.22 | 0.11 |
| Contralateral ICA occlusion | 1.65 | 0.06 – 47.47 | 0.74 |
| Diabetes mellitus | 0.30 | 0.07 – 1.16 | 0.09 |
| PAOD | 0.43 | 0.07 – 2.11 | 0.32 |
| SMD: absolute standardized mean difference. ^1^ 0: no collateral filling; 1: ≤50% but >0 of the occluded MCA territory; 2: >50% but <100% of the occluded MCA; 3: 100% collateral supply of the occluded MCA territory. CI: confidence interval; EVT: endovascular therapy; mRS: modified Rankin scale score; ICA: internal carotid artery; PAOD: peripheral arterial occlusive disease. | | | |

| **Table S8a:** Analysis of treatment effect on favorable outcome (mRS ≤2 after 90 days): multivariate conditional logistic regression of propensity score matched sample including all imbalanced covariates with SMD >0.1 | | | |
| --- | --- | --- | --- |
|  | **odds ratio** | **95% CI** | **p-value** |
| Univariate analysis |  |  |  |
| Treatment group EVT vs. BMT | 1.89 | 0.84 – 4.24 | 0.12 |
| Multivariate analysis |  |  |  |
| Treatment group EVT vs. BMT | 2.05 | 0.49 – 8.56 | 0.33 |
| Collateral score^1^ (n=70) | 2.00 | 0.40 – 10.03 | 0.40 |
| Arterial hypertension | 0.44 | 0.04 – 5.20 | 0.52 |
| Contralateral ICA occlusion | 1.03 | 0.04 – 27.73 | 0.99 |
| Diabetes mellitus | 0.55 | 0.08 – 3.88 | 0.55 |
| PAOD | 2.13 | 0.21 – 21.63 | 0.52 |
| SMD: absolute standardized mean difference. ^1^ 0: no collateral filling; 1: ≤50% but >0 of the occluded MCA territory; 2: >50% but <100% of the occluded MCA; 3: 100% collateral supply of the occluded MCA territory. CI: confidence interval; EVT: endovascular therapy; mRS: modified Rankin scale score; ICA: internal carotid artery; PAOD: peripheral arterial occlusive disease. | | | |

| **Table S9:** Sensitivity analysis with consideration of matched groups of mRS after 90 days by treatment groups (matched sample) | |
| --- | --- |
| **test** | **p-value** |
| For matching stratified Wilcoxon-Mann-Whitney test |  |
| Treatment group | 0.09 |
| Permutation test |  |
| Treatment group | 0.11 |
| For matching stratified Kruskal-Wallis test |  |
| Treatment group | 0.10 |
| mRS: modified Rankin scale sore | |

**Alternative matching models**

**Model 2: Inverse probability of treatment weighting (IPTW)**

**Figure S2:** Love plot of the absolute standardized mean differences (SMD) between endovascular therapy (EVT) and best medical treatment (BMT) before (raw) and after (matched) propensity score matching. Comparison of matching model 1 (main model) and model 3. ICA: internal carotid artery; mRS: modified Rankin Scale score; NIHSS: national institute of health stroke scale score; ASPECTS: Alberta stroke program early CT score.

**Model 1:** 1:1 nearest neighbor **propensity score matching** without replacement. Caliper 0.2, propensity score estimated with logistic regression, target estimand average treatment effect on the treated (ATT), number of observations 149 (raw) and 90 (matched). Covariates selected: NIHSS on admission, ipsilateral extracranial occlusion, age, hypercholesterolemia, sex, history of stroke, pre-stroke mRS and atrial fibrillation. 45 out of 75 original BMT cases were matched in pairs to 45 out of 74 original EVT cases. Balance of covariates after matching: 5/18 covariates with SMD >0.1. Mean SMD (unmatched): 0.216; mean SMD (matched): 0.111; reduction of mean SMD: 49%.

**Model 2:** **IPTW**. Propensity score of model 1, estimated with logistic regression, was used to calculate weights for subsequent analysis. Number of observations 149 (no exclusions). Covariates selected: Identical to model 1. Balance with weighting: 9/18 covariates with SMD ≥ 0.1. Mean SMD (unmatched): 0.216; mean SMD (weighted): 0.119; reduction of mean SMD: 45%.

Survey weighted generalized linear model, estimation of treatment effect: Odds ratio of EVT vs. BMT 1.88 (95%CI 0.89-3.96; p=0.10).

**Model 3: 1:1 nearest neighbor propensity score matching without replacement adding further covariate (diabetes mellitus)**

**Figure S3:** Love plot of the absolute standardized mean differences (SMD) between endovascular therapy (EVT) and best medical treatment (BMT) before (raw) and after (matched) propensity score matching. Comparison of matching model 1 (main model) and model 2. ICA: internal carotid artery; mRS: modified Rankin Scale score; NIHSS: national institute of health stroke scale score; ASPECTS: Alberta stroke program early CT score.

**Model 1:** 1:1 nearest neighbor **propensity score matching** without replacement. Caliper 0.2, propensity score estimated with logistic regression, target estimand average treatment effect on the treated (ATT), number of observations 149 (raw) and 90 (matched). Covariates selected: NIHSS on admission, ipsilateral extracranial occlusion, age, hypercholesterolemia, sex, history of stroke, pre-stroke mRS and atrial fibrillation. 45 out of 75 original BMT cases were matched in pairs to 45 out of 74 original EVT cases. Balance of covariates after matching: 5/18 covariates with SMD >0.1. Mean SMD (unmatched): 0.216; mean SMD (matched): 0.111; reduction of mean SMD: 49%.

**Model 3:** 1:1 nearest neighbor **propensity score matching** without replacement. Caliper 0.2, propensity score estimated with logistic regression, target estimand average treatment effect on the treated (ATT), number of observations 149 (raw) and 90 (matched). Covariates selected: Same as model 1 with additional diabetes mellitus. 45 out of 75 original BMT cases were matched in pairs to 45 out of 74 original EVT cases. Balance of covariates after matching: 9/18 covariates with SMD >0.1. Mean SMD (unmatched): 0.216; mean SMD (matched): 0.100; reduction of mean SMD: 54%.

Estimation of treatment effect in model 3 with conditional logistic regression: Odds ratio of EVT vs. BMT 2.14 (95% CI 0.87-5.26; p=0.10).

References:

1. Ho D, Imai K, King G, et al. MatchIt: Nonparametric Preprocessing for Parametric Causal Inference. *Journal of Statistical Software* 2011;42:1-28. doi: 10.18637/jss.v042.i08

2. Nguyen T-L, Collins GS, Spence J, et al. Double-adjustment in propensity score matching analysis: choosing a threshold for considering residual imbalance. *BMC Medical Research Methodology* 2017;17(1):78. doi: 10.1186/s12874-017-0338-0

3. Westreich D, Greenland S. The Table 2 Fallacy: Presenting and Interpreting Confounder and Modifier Coefficients. *American Journal of Epidemiology* 2013;177(4):292-98. doi: 10.1093/aje/kws412
